# The characterization of the circadian clock in the olive fly *Bactrocera oleae* (Diptera: Tephritidae) reveals a *Drosophila*-like organization

**DOI:** 10.1038/s41598-018-19255-8

**Published:** 2018-01-16

**Authors:** Enrico Bertolini, Christa Kistenpfennig, Pamela Menegazzi, Alexander Keller, Martha Koukidou, Charlotte Helfrich-Förster

**Affiliations:** 1 Neurobiology and Genetics, Theodor Boveri Institute, Biocentre, University of Würzburg, 97074 Würzburg, Germany; 2 Oxitec Ltd, 71 Milton Park, Oxford, OX14 4RQ UK; 3 Center for Computation and Theoretical Biology and Department of Bioinformatics, Biocentre, University of Würzburg, 97074 Würzburg, Germany

## Abstract

The olive fruit fly, *Bactrocera oleae*, is the single most important pest for the majority of olive plantations. Oxitec’s self-limiting olive fly technology (OX3097D-Bol) offers an alternative management approach to this insect pest. Because of previously reported asynchrony in the mating time of wild and laboratory strains, we have characterized the olive fly circadian clock applying molecular, evolutionary, anatomical and behavioural approaches. Here we demonstrate that the olive fly clock relies on a *Drosophila melanogaster*-like organization and that OX3097D-Bol carries a functional clock similar to wild-type strains, confirming its suitability for operational use.

## Introduction

The fruit fly *Bactrocera oleae* (Rossi) (Diptera: Tephritidae) is a major pest of cultivated olives. Its presence has been historically reported in Mediterranean and African countries and, more recently, it has spread to Central America and California^1^. The adult female fly lays approximately 800 eggs during its lifetime, through the skin of olive fruits. The hatching larvae feed while tunneling in the fruit mesocarp causing crop damage and premature drop. Multiple and overlapping generations every year cause enormous loss to olive agriculture and the economy of olive products^2^. Current control methods against olive fly rely overwhelmingly on the use of chemical insecticides. Several insecticides have been, or are being, phased out due to concerns about their negative impact on the environment or human health. This reduces control options and increases the rate at which the olive fly becomes resistant to the remaining insecticides^3^.

The Sterile Insect Technique (SIT) is a targeted method of pest control that involves the mass rearing and release of sterilized insects (traditionally by irradiation) into the wild. The introduction of an excess of sterile insects reduces the reproductive potential of the target population through infertile matings, leading to suppression^4–6^. Due to its high economic burden and the over-use of chemical control, the olive fly was among the first insects to be considered for SIT. However, previous attempts using irradiated mixed-sex insects achieved very limited success, due to the poor quality of the irradiated insects and mating asynchrony between the laboratory-reared and wild olive flies^7–9^. “Self-limiting” engineered olive fly strains can unlock the SIT potential for the control of this economically important agricultural pest^10^.

The success of SIT, whether traditional or using novel technologies, is largely driven by the mating behaviour of the insects of concern. The mass-released males must exhibit the same mating behaviour as wild males, or they may be unsuccessful in gaining females^11^. In most *Bactrocera* species mating occurs at a species-specific time window during the day, most commonly associated with dusk and less frequently midday^12^, at which time male sexual activity has to be synchronous with female receptivity^13^. In *Bactrocera*, this timing of mating behaviour is known to be modulated by the circadian clock^14,15^, as it is in *Drosophila*^16^. It has been shown that in *Bactrocera* this can be very sensitive to insect colony adaptation in laboratory conditions^17^.

Yet, very little is known about the mechanisms of the circadian clock in *Bactrocera* species (Tephritidae); in contrast to the fruit fly, *Drosophila melanogaster* (Drosophilids), that has been widely adopted as the primary insect model. *D. melanogaster* possesses a molecular clock which relies on the interaction between several clock genes and their respective products. Two main transcription/translation feedback loops (TTFLs) drive single cell molecular oscillations which are at the basis of circadian physiology and behaviour^18^. A first loop involves the clock genes *period* (*per*), *timeless* (*tim*), *Clock* (*Clk*) and *cycle* (*cyc*). A second loop involves the clock genes *Par-domain-protein-ε* (*Pdp1ε*) and *vrille* (*vri*) that interlock with the first loop regulating the expression of *Clk*. Under light-dark cycles, the molecular oscillation is reset every day via the blue-light photoreceptor cryptochrome (CRY)^19,20^.

The master pacemaker in *Drosophila* is located in the Central Nervous System (CNS), where around 75 clock neurons per brain hemisphere drive synchronous oscillations in seven distinct neural cluster (s-LN_v_, l-LN_v_, LN_d_, LPN, DN_1_, DN_2_, DN_3_). The clock network uses neuropeptides like Pigment Dispersing Factor (PDF)^21,22^ and Ion Transport Peptide (ITP)^23,24^ to coordinate the oscillations in the different clusters. This complex network supports rhythmicity in locomotor activity under cycles of alternated light and dark (LD) and constant darkness (DD), whereas it is lost under constant light (LL)^25^.

Circadian rhythms in *B. oleae* were previously described for several behaviours (i.e. mating^26^, exodus of larvae from diet^27,28^ and pheromone emission^29^). Here we characterize the circadian clock of *B. oleae* at molecular, evolutionary, anatomical, and behavioural levels, and we show that the olive fly carries a *Drosophila*-like clockwork in all of these aspects. Furthermore, we demonstrate that the self-limiting olive fly strain OX3097D-Bol possesses a functional circadian clock that does not differ from wild-type genotypes.

## Results

### Identification of *B. oleae* clock genes

We identified the whole length transcript sequences of the clock genes *per*, *cyc* and *cry*, as well as partial sequence of *Clk* (Table 1).

**Table 1:** Clock genes identified from *Bactrocera oleae* (and see Fig. 1).

Gene	Length	Putative protein coding and other details
*per* CDS	3105 bp	A protein of 1034 aa residues, containing two Nuclear Localization Signals (NLS, aa 72 to 78 and 691 to 718), two PER-ARNT-SIM domains (PAS-A, aa 193 to 242; PAS-B, aa 343 to 395) and a Cytoplasmic Localization Domain (CLD, aa 398 to 457). There is only a single Threonine-Glycine tandem repeat (T-G) in the *B. oleae* PER sequence (Supplementary Fig. S1)
*cyc* CDS	1206 bp	A 401 aa polypeptide containing a basic Helix-Loop-Helix motif (bHLH, aa 22 to 74) followed by PAS-A (aa 114 to 157) and PAS-B (aa 306 to 353) domains (Supplementary Fig. S2)
*cry* CDS	1644 bp	A 547 aa protein, in which the DNA photolyase activity domain (aa 4 to 195) and the FAD binding domain (aa 213 to 516) contains the conserved putative interaction sites for FAD (Flavin Adenine Dinucleotide; 13/14 identity), MTHF (methenyltetrahydrofolate; 7/7 identity) and CPD (cyclobutane pyrimidine dimers; 14/14 identity) (Supplementary Fig. S3)
*Clk* (5′*Clk* CDS)	990 bp	A 330 aa truncated peptide that contains most of the functional domains, such as bHLH (aa 13 to 58) and PAS (PAS-A, aa 92 to 140; PAS-B, aa 252 to 297), and has high sequence homology to *Clk* of *D. melanogaster* (Supplementary Fig. S4).

**Figure 1:**
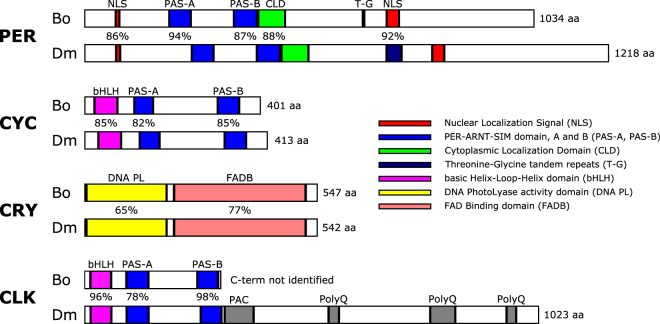
Schematic representation of the predicted structure of *B. oleae* PER, CLK, CYC and CRY. *B. oleae* sequences (top) are compared to their respective *D. melanogaster* counterparts (bottom). Coloured parts are the functional domains for which the percentage of identity between the two sequences is indicated. Rectangle length corresponds to sequence length; the entire length of the proteins is indicated at the right end. An identification key for the different domains is present in the figure. Multiple sequence alignment and a more detailed overview of proteins structure can be found in Supplementary Figures S1–S4.

**Figure 2:**
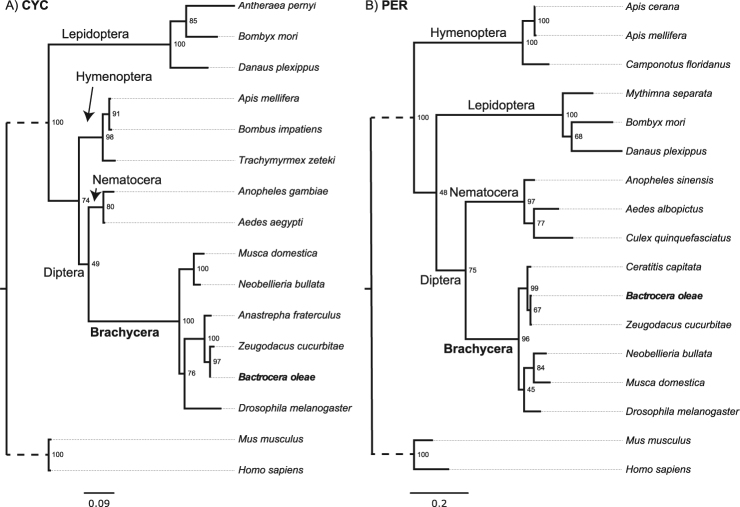
Phylogenetic analysis of *B. oleae* PER and CYC. Gene trees of (**A**) CYC and (**B**) PER reconstructed from amino acids with Maximum Likelihood using RaxML. Values at the nodes represent bootstrap values, determined with 1000 replicates.

When aligned with their respective *Drosophila* and *Bactrocera* counterparts, the predicted amino acid sequences show high identity levels (Supplementary Figs S1–S4), particularly within the characterized functional domains (Fig. 1). However, other Diptera such as mosquitoes and sandflies (Nematocera) carry the mammalian form of *cyc*, *cry* and *Clk* (see Discussion). The overall high similarity between *Bactrocera* and *Drosophila* clock genes, especially for *cyc*, *cry* and *Clk*, suggests that the molecular clock of the two genera is very similar, but clearly different from the clock of other insects (Fig. 2).

Our phylogenetic analysis also suggests that the evolutionary pace of substitutions for the Brachycera branch was by far higher than expected regarding the remaining tree for the *cyc* gene (χ^2^ = 73.24, df = 1, p < 0.001), and slightly for *per* (χ^2^ = 5.01, df = 1, p < 0.05). This supports the notion of dramatic changes in the evolutionary history of the clock genes in this group in comparison to other insects.

### Temporal expression of clock genes in *B. oleae* heads

**Figure 3:**
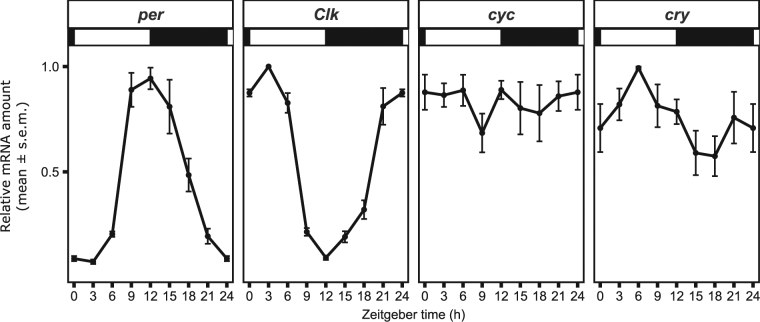
Temporal expression of the clock genes *per*, *Clk*, *cyc* and *cry* in *B. oleae* heads. In Argov male flies, *per* and *Clk* mRNA levels are strongly rhythmic and in antiphase to each other (*per*: H(7) = 20.9, p = 0.003; CircWave, p < 0.001 – *Clk*: H(7) = 21.2, p = 0.003; CircWave, p < 0.001). The gene *cyc* is constantly expressed (H(7) = 4.1, p = 0.771; CircWave, p > 0.05). *cry* mRNA shows weak oscillation (H(7) = 11.6, p = 0.115; CircWave, p < 0.001).

In order to see whether the TTFLs of *B. oleae* run similar to those of *D. melanogaster*, we analysed the temporal expression of *per*, *Clk*, *cyc* and *cry* under light-dark cycles of 12 h:12 h (LD12:12). As reported previously for *D. melanogaster*^30,31^, we detected strong and significant circadian oscillations in *per* and *Clk* mRNA levels that were in anti-phase to each other, with their respective peaks at Zeitgeber time (ZT) 12 and ZT3 and troughs at ZT0 and ZT12 (Fig. 3). In contrast, the clock gene *cyc* was constantly expressed as shown in *D. melanogaster*^32^. Furthermore, similar to *D. melanogaster*^19^, we observed weak circadian oscillations in *cry* mRNA levels of *B. oleae*, with a peak around ZT6 and a trough around ZT18 (Fig. 3). For comparison, *cyc* is known to oscillate in anti-phase to *per* in other insects and mammals, whereas *Clk* is constantly expressed (see Discussion). Taken together, our results show that the regulation of the expression of *B. oleae* clock genes relies on mechanisms common to the *D. melanogaster* TTFLs.

**Figure 4:**
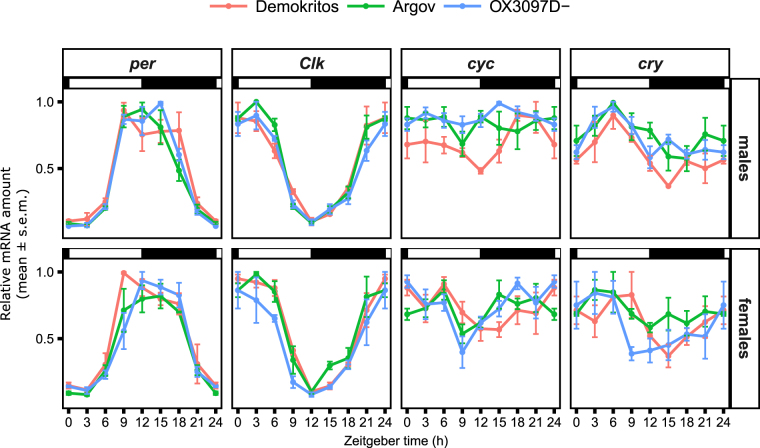
Comparison of clock genes expression between wild-type and transgenic *B. oleae*. Oscillations in the expression of *per*, *Clk*, *cyc* and *cry* under LD12:12 was investigated in three different genotypes: Demokritos, Argov (wild-type) and OX3097D-Bol. No significant interaction among timepoint and genotype was found using 2-way ANOVA (all p-values > 0.05, see Supplemetal Table 1), indicating no differences in amplitudes nor phases among strains.

Most importantly, when the expression levels of these genes were compared among wild-type and genetically engineered strains (Fig. 4), we found no significant differences in phases or in amplitude (Supplementary Table S1), demonstrating that the TTFLs of the strain OX3097D-Bol do not differ from the wild-type strains in their regulation of clock gene expression.

### The clock network in *B. oleae* brain

**Figure 5:**
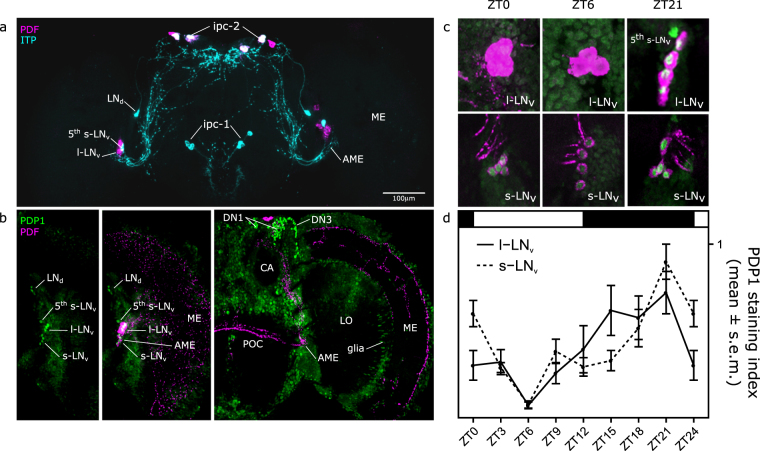
The clock network of *B. oleae*. (**a**) The clock neuropeptide PDF (magenta) is expressed in 4 l-LN_v_ and 4 s-LN_v_, as well as in 4 putative insulin producing cells (ipc-2) in the PL. ITP (cyan) is expressed in the 5^th^ s-LN_v_ and in one cell of the LN_d_ group, as well as in putative ipc-1 and ipc-2. (**b**) anti-PDP1 (green) co-localize in the nuclei of PDF positive cells (magenta) in the LN_v_ cluster. Antibody reveals also other putative clock clusters (LN_d_, DN), and stains many other non-clock cells. (**c**,**d**) PDP1 protein level in the nuclei oscillates under LD12:12 in the s-LN_v_ (H(7) = 34.78, p < 0.001) and l-LN_v_ (H(7) = 19.259, p < 0.05). AME: accessory medulla; ME: medulla; CA: calyx; LO: lobula.

We first characterized the neuroarchitecture of the *B. oleae* clock network by investigating the expression pattern of the clock neuropeptides PDF, ITP and the core clock protein PDP1 (Fig. 5a–c). We identified four small (s-LN_v_) and four large (l-LN_v_) ventro-Lateral-Neurons that express PDF, as well as four neurosecretory cells (putative IPCs, Insulin-Producing-Cells; we call them ipc-2 cells according to the naming in *D. melanogaster*) in the pars lateralis (PL).

The 4 s-LN_v_ project to the dorsal protocerebrum, whereas the 4 l-LN_v_ innervate the medulla and project to the contralateral hemisphere via the posterior optic commissure (POC) (Fig. 5a,b). ITP is expressed in the 5^th^ s-LN_v_ and in one cell of the dorsal Lateral Neurons (LN_d_), as well as in three of the four PDF positive ipc-2 cells in the PL. ITP is additionally stained in three other putative IPC cells (ipc-1 as they are named in *D. melanogaster*) in the central brain. When we perfomed double-staining using anti-PDF and anti-PDP1 antibodies together, cytoplasmic PDF signal co-localized with nuclear PDP1 signal in both small and large clusters, confirming the homology with *D. melanogaster* lateral ventral clock neurons (Fig. 5c). In addition, anti-PDP1 antibody labeled also the other clock clusters (DN_1_, DN_3_, LN_d_) that were located in a similar position as the homologous neurons in different *Drosophila* species^33^. In order to see whether PDP1 oscillates in its abundance, we analysed the staining intensity of PDP1 in the LN_v_ (co-labelled with PDF) at several timepoints across the LD12:12 cycle. We found synchronous oscillations in the levels of PDP1 in both s-LN_v_s (H(7) = 34.78, p < 0.001) and l-LN_v_s (H(7) = 19.26, p < 0.05) (Fig. 5d), reaching their maxima and minima at ZT21 and ZT6, respectively. These oscillations are similar to the oscillations of PDP1*ε* described in *D. melanogaster*^34^.

### Locomotor activity rhythm of *B. oleae*

**Figure 6:**
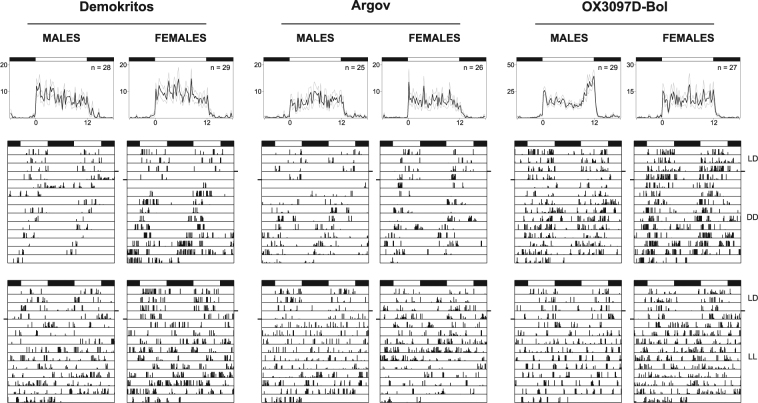
Locomotor activity of *B. oleae* under different conditions. Daily activity profile under LD12:12 (top) and one representative actogram, respectively under DD (middle) and LL (bottom) of males (right) and females (left) of Demokritos, Argov and OX3097D-Bol at 20 °C. Horizontal bars aside the actograms separate LD and DD/LL phases.

In order to determine the endogenous clock properties of wild-type and self-limiting strains, we recorded locomotor activity as a readout of the circadian pacemaker activity. In general, activity recording worked well in olive flies, although they showed a high mortality rate when isolated into the glass tubes (~50% of the flies died within the first week). Furthermore, olive flies showed low activity levels (on average 17 beam crosses/hours, Supplementary Fig. S5). Under LD12:12 cycles, all flies were diurnal and more or less uniformly active throughout the day (Fig. 6). Only OX3097D-Bol males were significantly more active (on average 39 beam crosses/hours; H(5) = 56.17, p < 0.001; Supplementary Fig. S5) and exhibited bimodal activity, with more activity in the morning and evening as described for *D. melanogaster* (Fig. 6). The amount of activity associated with the evening was significantly different among strains (F(5) = 6.8, p < 0.001), with OX3097D-Bol males showing the highest level (Supplementary Fig. 6).

**Figure 7:**
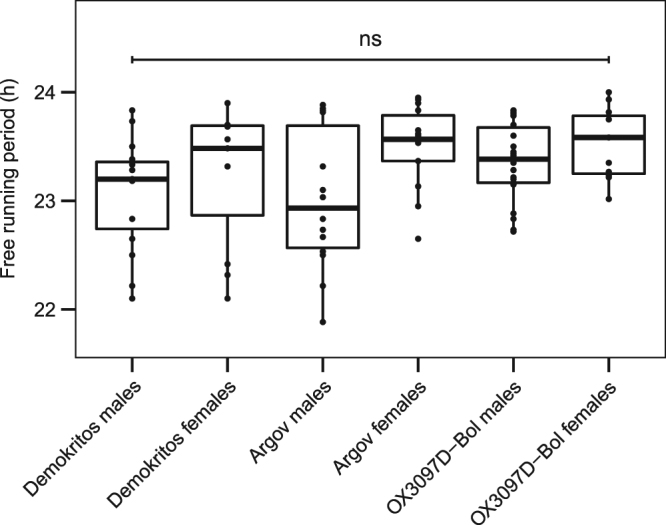
Average free running periods of the different strains of *B. oleae*. No significant differences in the free-running periods of males and females of Demokritos, Argov and OX3097D-Bol strains are found under DD (H(5) = 10.92, p > 0.05).

After transfer in DD, the majority of flies showed self-sustained rhythmicity in their locomotor activity, with a free-running period shorter than 24 hours in all genotypes tested (Figs 6 and 7 and Table 2). No significant differences were found in the percentage of rhythmic flies (Table 2; χ^2^ = 4.8, df = 5, p = 0.44) and in the free-running period (Fig. 7, H(5) = 10.92, p = 0.053) between the different strains and sexes. When flies were released in constant light (LL), their locomotor activity became arrhythmic as known to happen in *D. melanogaster* (Fig. 6 and Table 2).

**Table 2:** Free running period and rhythmicity percentage of olive flies under DD and LL.

genotype	gender	DD		LL
		% rhythmic flies (N)	period ± s.e.m. (h)	% rhythmic flies (N)
**Demokritos**	M	72.7 (22)	22.99 ± 0.15	0 (16)
	F	91.7 (12)	23.24 ± 0.19	0 (26)
**Argov**	M	73.7 (19)	23.01 ± 0.17	0 (15)
	F	93.3 (15)	23.49 ± 0.1	0 (12)
**OX3097D-Bol**	M	78.6 (28)	23.36 ± 0.08	0 (14)
	F	68.8 (16)	23.54 ± 0.1	0 (20)

Taken together, these results demonstrate that locomotor activity of *B. oleae* is clock-controlled and that the fundamental clock properties in the olive fly are common with *D. melanogaster*.

## Discussion

### The molecular clock of *B. oleae*

Although the fruit fly *Drosophila melanogaster* has been of exceptional importance in understanding how the molecular clock in an insect model ticks, studies in other insects with partially annotated genomes highlight differences in the underlying molecular mechanisms. Striking differences in the structure and expression of some clock genes are already evident among flies (order Diptera).

For example, Nematocera (lower Diptera, such as mosquitoes or sandflies) have been shown to carry the mammalian form of the gene *cyc*^35^, also called Bmal1 (Brain and muscle ARNT-like). This alternative form encodes a peptide that carries an extra domain at the C-terminus (BCTR, BMAL C-terminus region) working as transactivation domain required for binding and activating transcription of the *per* gene. Most probably because of this, *cyc* is rhythmically expressed in mosquitoes^35,36^, whereas in *Drosophila* and other higher Diptera (=Brachycera) it is constantly expressed^32,37,38^. Here we report that *B. oleae cyc* shares the same features as *cyc* in other Brachycera flies.

In higher Diptera, the CLK protein possesses a transactivation domain (multiple poly-Q sites at the C-terminus) and is responsible of activating *per* transcription and oscillates in abundance^39^. The CLK protein of lower Diptera (mosquitoes and sandflies) is considerably shorter than in higher Diptera and neither oscillates in abundance nor binds and activates *per*^35,36^. Since we could only isolate the 5′*Clk* CDS of *Bactrocera*, we do not know whether *Clk* possesses the poly-Q sites typical for *D. melanogaster Clk*. Nevertheless, we found that *Clk* mRNA cycles in abundance, making it very likely that the transactivation domain is present.

Most likely, the CYC and CLK found in lower Diptera are the ancestral forms that are conserved in most insects and mammals. During evolution, the higher Diptera (Brachycera) seem to have lost the BCTR activation domains at the C-terminus of CYC but gained the poly-Q repeats at the C-terminus of CLK (see^38^ for discussion). *Bactrocera oleae*, belonging to the higher Diptera (Brachycera; family Tephritidae), fits perfectly into this picture.

Like *cyc* and *Clk*, the gene *cryptochrome* is also found in two distinct variants in Diptera: *cry1* and *cry2*. *Cry1* is the only cryptochrome present in *Drosophila*; it is light sensitive and acts as the main circadian photoreceptor in *Drosophila’s* master clock^19,20^. In contrast, *cry2* is the mammalian form of cryptochrome; it is not light-sensitive, and it is instead involved in the core feedback loop^40,41^. Nematocera, for example mosquitoes, express both types of cryptochrome. In *B. oleae* we have identified *cry1*, but not *cry2*: the sequences annotated in GenBank as *Bactrocera cry2* are phylogenetically closer to photolyases rather than to the mammalian-like *cryptochrome* (see Supplementary Fig. S7). A similar situation occurred already in *Musca domestica*, where Bazalová and Dolezel^42^ found that the gene previously annotated as *cry2* actually belongs to photolyases. Most importantly, *B. oleae cry1* carries all the domains needed for binding the chromophores flavin and MTHF, indicating that it is likely to work as a circadian photoreceptor. This feature is also confirmed by the arrhythmic activity of *Bactrocera* flies under LL. In addition, our findings fit in with the results obtained from other *Bactrocera* species, such as *B. tryoni*, *B. neohumeralis* and *Z. curcubitae*^43,44^.

The *per* gene has been characterized in many insect species including some tephritid fruit flies (*Bactrocera* and *Ceratitis* species)^45–48^. In all these species, *per* carries the same structure and functional motifs and exhibits daily rhythmic expression as in *D. melanogaster*. Here we confirmed that also *B. oleae per* shares the same features.

In other insect orders, such as the Lepidoptera and Hymenoptera for example, the clock scenario gets more complex. Nevertheless, all investigated species so far express the mammalian form of CYC, CLK and CRY^40,49–51^. In contrast, the overall picture shows that *B. oleae* carries clock components that are of the *Drosophila* type (perhaps better Brachycera type), and their expression under light-dark cycles is highly coherent with the respective *Drosophila* profiles. Our phylogenetic analysis on *B. oleae* PER and CYC highlights how the clock of Brachycera species might be exceptional among insects, and that a molecular clockwork different from the one of *Drosophila* might be the most common model among Hexapoda.

### The clock network in the brain

At the anatomical level, several studies have shown that the distribution of certain neuropeptides in the clock network are quite different within closely related species in the Brachycera. For example, *Drosophila* species that inhabit cold habitats (high latitudes) differ in their PDF expression pattern from *D. melanogaster*: They lack PDF in the s-LN_v_^33,52,53^. Here we show that the olive fly, like *D. melanogaster*, expresses PDF in both s-LN_v_ and l-LN_v_. Additionally, we demonstrate that the expression of the other central clock neuropeptide, ITP^23,24^, is highly conserved.

Anti-PDP1 antibody was previously used to reveal the clock neurons in several *Drosophila* species^33^. It worked also in *B. oleae* and stained neurons in the lateral and dorsal brain that appear homologous to those seen in all *Drosophila* species tested so far. Furthermore, PDP1 cycled in a circadian fashion in the s-LN_v_ and l-LN_v_ as reported for *D. melanogaster*^34^.

We conclude that the clock network in the brain of *B. oleae* is highly similar to that of *D. melanogaster;* even more similar than the clock network of *Drosophila* flies from higher latitudes. *B. oleae* is prevalent in warm habitats mostly around the Mediterranean area. This underscores our hypothesis that changes in the clock network preferentially happened in species that radiated to the north, but not in flies that remained in subtropical regions^53^. We are not aware of any study performed on the clock network in the brain of lower Diptera. To further prove this hypothesis, it would be most interesting to investigate the PDF-pattern in lower Diptera, such as mosquitoes, of which species are found at all latitudes ranging from tropical regions to the very north or south.

### Behavioural rhythms of *B. oleae*

Despite the high mortality rate and low activity of the isolated flies, we could clearly show that *B. oleae* locomotor activity is under the control of the circadian clock: rhythmic locomotor activity pattern persisted in the absence of all *Zeitgebers* under constant darkness (DD). We suspect that the relative low activity level of olive flies might be due to their natural life style. In the wild, *Bactrocera* flies (especially males) spend much of the day resting in the canopy of trees^54^, with resource foraging and mating behaviours at temporally distinct times^55^. Laboratory mass rearing is known to further decrease the tendency of flies to move^56^. The lack of natural environmental stimuli in the glass tubes may also lead to the observed low activity. Nevertheless, the activity level of the majority of flies was high enough to reveal a circadian rhythm in locomotor activity under DD conditions. This is consistent with our finding that PDF is present in the s-LN_v_. The latter has been shown to be essential for robust activity rhythms under constant darkness^53^. Fly species lacking PDF in the s-LN_v_ show arrhythmic activity under DD conditions^52,53,57,58^. In contrast to DD conditions, *B. oleae* flies were arrhythmic under constant light. The same is observed in *D. melanogaster* and can be explained by the permanent degradation of the clock protein TIM via the light-sensitive CRY1^59^. The same model seems to apply to *B. oleae*.

Taken together, our results clearly depict, from three different points of view (molecular, anatomical and behavioural), that the general organization of the *B. oleae* circadian clock highly matches the well known clock of *D. melanogaster*. For this reason, we propose that the molecular basis of the olive fly clock relies on a *Drosophila*-like mechanism.

### Impact of our study on the application of SIT method against *B. oleae*

One relevant aspect of our study was the investigation of the self-limiting strain, OX3097D-Bol, which offers an alternative olive fly pest control strategy to chemical interventions. Despite the continued broad use of pesticides to keep insect pest populations under control, reduced or eliminated pesticide use is getting the consensus of growers and consumers as the more sustainable way to manage pest species in agriculture. Among these, SIT is already applied in modern agriculture to fight several Tephritidae species such as the Mediterranean fruit fly (*Ceratitis capitata*), the Oriental fruit fly (*Bactrocera dorsalis*), the Melon fly (*Bactrocera cucurbitae*) and the Mexican fruit fly (*Anastrepha ludens*)^60–63^. Unexpectedly, this method has proven to be inefficient in the control of *B. oleae* in the past. One of the reasons suggested as a cause for the low success was the mating asynchrony reported between wild and laboratory-reared individuals^8,9^. In these studies, the possible role of the circadian clock was not taken into account, and it was for this reason that we examined the circadian clock of the self-limiting olive fly strain OX3097D-Bol. We now clearly demonstrate that OX3097D-Bol expresses the clock genes in the same temporal manner as wild-type flies. Additionally, our behavioural studies show that the endogenous period of this strain does not differ from the wild-type genotypes. Together, this supports the fact that the circadian clock of OX3097D-Bol is functional and unaltered from the wild-type strains.

Interestingly, OX3097D-Bol showed higher overall locomotor activity, especially in the evening, compared to wild-type flies. These increased evening activity levels overlap with the timing of mating, which is known to occur in the hours prior to scotophase both under natural and artificial conditions^26^. Whether this may be advantageous to future operational applications is not investigated here. However, in other *Bactrocera* species, increased male activity at time of mating has been directly linked to increased male mating success^64^. We thus speculate that increased locomotor activity of OX3097D-Bol males during the time window of female receptivity may increase mating success. In conclusion, our results suggest that the clock structure and expression remain intact in the self-limiting olive fly strain OX3097D-Bol, and together with previously published data^10^ we conclude that sustained release of OX3097D-Bol males can be a viable stategy for olive fly pest control.

## Material and Methods

### Fly strains and husbandry

The olive fly strains considered in this article are the following: the wild-type strains Argov^65^ and Demokritos^66^ and the self-limiting strain OX3097D-Bol^10^. The self-limiting strain of *B. oleae*, OX3097D-Bol, incorporates a tetracycline-off system designed to produce a conditional female-specific self-limiting trait when reared in the absence of a sufficient concentration of tetracycline. The OX3097D-Bol also expresses a fluorescent marker gene to enable detection and monitoring in operational deployment. Flies were reared on standard olive fly larval and adult diet and maintained under cycles of 12 hours of light followed by 12 hours of darkness (LD12:12) at 23°C (±2 °C) and 50% humidity (±10%)^10^.

### Cloning of clock genes

Wild-type flies were collected in dry ice, and RNA was isolated from heads using the Total RNA Purification Kit (Norgen Biotek). cDNA was obtained by reverse transcription using oligo-dT primers and the RevertAid First Strand cDNA Synthesis Kit (Thermo Fisher Scientific). A PCR-based strategy using degenerate oligonucleotide primers designed over highly conserved regions of reported *Drosophila* and *Bactrocera* clock genes was used to isolate the olive fly homologous of *per*, *cry*, *Clk* and *cyc* (see primer sequences in SupplementaryTable S2). The same strategy was initially used for the isolation of *timeless*, which has also been annotated in the genome of *B. oleae* (XM_014247668.1). Nevertheless, for technical reasons we decided not to pursue the charachterization of this gene any further. 3′ and 5′ RACE PCRs were used to obtain the full-length cDNA sequence (SMARTer RACE cDNA Amplification Kit, Clontech Laboratories). PCRs were performed in a T3000 Thermal Cycler (Biometra) using Q5 Hot Start Polymerase (NEB). PCR products were purified either directly from the reaction (QIAquick PCR Purification Kit, QIAGEN) or from agarose gel (QIAquick Gel Extraction Kit, QIAGEN), and cloned into pJet1.2/blunt vector (Thermo Fisher Scientific). Positive clones were identified by colony PCR screening and purified plasmids (GeneJET Plasmid Miniprep Kit, Thermo Fisher Scientific) were sent to sequencing (GATC Biotech).

### Phylogenetic analysis

A phylogenetic analysis was conducted for each of the genes of interest, to infer information about the evolutionary background of the genes. For this, sequences of *cyc* and *per* were respectively aligned using ClustalOmega^67^ with corresponding reference sequences obtained from GenBank^68^ from other insect taxa, and as the outgroup for the analysis human and mouse (GenBank identifiers in Supplementary Table S3). Only homologous parts of the sequences in at least 50% of the sequences were kept, the rest masked out using GBlocks^69^ to avoid too much divergency for phylogenetic interference. This clean alignment for each of the genes was then used for a phylogenetic reconstruction using RaxML v8.0.0^70^ with 100 bootstraps and the BLOSUM62 substitution matrix. To test whether the Brachycera branch of each phylogeny corresponded to the overall substitution rates of the corresponding gene tree or evolved with a different evolutionary speed, PAML^71^ was used (mammals excluded). For this we estimated the log likelihood (lnL) of the null-model with a fixed rate over all branches and one model with a released rate for the Brachycera branch for each gene tree. The differences between both models were compared with χ^2^ = 2* (lnL_nullmodel_ − lnL_model_) and the statistical significance inferred with a χ^2^ table.

### Real time PCR and analysis

From the late pupal stage onwards, flies were entrained under LD12:12 (23 °C, 60% humidity) and 3 to 5 days individuals were sampled separately. Total RNA was isolated from heads (4 heads/sample) and cDNA generated as described in the previous paragraph. Experiments were carried out using a thermal cycler (MX3005P, Stratagene) and reverse transcription products were detected using a DNA-binding fluorescent dye (SYBR Green PCR Master Mix, Applied Biosystems) following the manufacturer’s protocol. Thermal cycling consisted of 10 minutes incubation at 95 °C, followed by 40 cycles of denaturation (30 seconds at 95 °C), annealing (1 minute at 60 °C) and extension (1 minute at 72°C) followed by melting curves (55 °C–95 °C). For each time series, reactions were set up in duplicates on one 96-well plate, including negative controls (-cDNA) for each gene. Relative clock gene mRNA abundance was normalized against the constantly expressed reference gene 17 S rRNA transcript and analyzed using the ddCt method.

### Immunocytochemistry and staining quantification

Flies were sampled in 4% paraformaldehyde (PFA) in Phosphate Buffer Saline with the addition of Triton-X100 at the concentration of 0.1% (PBST) and fixed for 4 hours at room temperature. PFA was removed by several washes of PBS, and right afterwards brains were manually dissected. Blocking solution (5% Normal Goat Serum (NGS) in PBST) was applied for 4 hours at room temperature and afterwards samples were transferred in primary antibodies solution for at least one day at room temperature. Primary antibodies were diluted in PBST with 5% NGS and 0.02% NaN_3_. Those used in this work are the following: mouse anti-PDF c7 (1:500, DHSB), rabbit anti-PDF-cricket (1:1500^72^); rabbit anti-ITP (1:10000^73^); rabbit anti-PDP1 (1:1000^74^). After incubation, primary antibodies were removed from the tissue by washes with PBST and secondary antibodies (anti-mouse or anti-rabbit, Alexa Fluor 488, Alexa 555 or Alexa Fluor 635 conjugated; 1:400 in PBST with 5% NGS) applied overnight at 4 °C. Finally, samples were washed with PBS and mounted on slides using Vectashield medium (Vector Laboratories). For the double labelling with anti-PDP1 and anti-PDF antibodies (Fig. 4), which are both raised in rabbit, the full staining procedure was performed twice. The first anti-PDP1 staining (labelled with anti-rabbit Alexa Fluor 488) was fixed a second time and the protocol repeated using anti-PDF-cricket antibody (labelled with anti-rabbit Alexa Fluor 635). Confocal images were acquired every 2 μm Z-stacks using a Leica TCS SPE (Leica). The quantification of PDP1 levels was performed measuring the staining intensity of PDP1 in the nuclear region of PDF positive cells using the software FIJI^75^. Thereafter, background fluorescence was substracted from the mean intensity of each cluster and final values were averaged among hemispheres.

### Locomotor activity recording and analysis

Adult flies (5 to 10 days after eclosion) were placed in single locomotor activity tubes (10 cm length, 1 cm diameter) where diet was supplied on one side as 4% saccharose and 2% agarose in water. Locomotor activity was recorded using a custom monitoring device (TriKinetics Inc) as the number of times that an infrared light beam crossing the middle of the tube was interrupted by a passing fly^76^. Monitors were placed in incubators (Panasonic, MIR-154-PE) where temperature was kept constant at 20 °C and flies were entrained to artificial square cycles of 12 hours of light and 12 hours of dark (LD12:12, approximately 200 lux). Constant dark and constant light was applied after entrainment at the same constant temperature.

Activity was recorded every minute; later the dataset was binned in 15 minute-intervals to get rid of noisy and erratic activity bouts in recordings. The daily profile of locomotor activity has been calculated on manually selected days for each single flies.

The total amount of daily activity was calculated as the sum of beam crosses counted during the light phase in each single fly and averaged across all insects recorded under the same condition. The relative amount of evening activity was calculated using the same procedure considering only the last part of the day (from ZT9 to ZT12) and dividing it by the total activity.

Representative double-plotted actograms in this paper show the last four days of entrainment followed by 10 days of constant darkness (DD) or constant light (LL).

The free-running period in DD has been determined on selected consecutive days for each single individual using the Lomb-Scargle periodogram implemented in the ImageJ plugin ActoJ^77^. Flies were considered rhythmic judged on both visual inspection and significance of the Lomb-Scargle periodogram (p < 0.05).

### Statistical analysis

Statistical analysis was performed using R software. Normal distribution of samples was tested using the Shapiro test. Equality of variances among samples was tested using Levene’s test. According to the outcome, 1-way ANOVA, 2-way ANOVA or Kruskal-Wallis H tests was applied. Pairwise comparisons between group levels were corrected for multiple testing using the Bonferroni method. To detect cycling in the daily gene expression profiles, we additionally used the software CircWave^78^.

### Data Availability

The datasets generated during and/or analysed during the current study are available from the corresponding author on request.

## Electronic supplementary material

Supplementary Information
