# Author Correction: The characterization of the circadian clock in the olive fly *Bactrocera oleae* (Diptera: Tephritidae) reveals a *Drosophila*-like organization

**DOI:** 10.1038/s41598-018-24850-w

**Published:** 2018-04-30

**Authors:** Enrico Bertolini, Christa Kistenpfennig, Pamela Menegazzi, Alexander Keller, Martha Koukidou, Charlotte Helfrich-Förster

**Affiliations:** 1 Neurobiology and Genetics, Theodor Boveri Institute, Biocentre, University of Würzburg, 97074 Würzburg, Germany; 2 Oxitec Ltd, 71 Milton Park, Oxford, OX14 4RQ UK; 3 Center for Computation and Theoretical Biology and Department of Bioinformatics, Biocentre, University of Würzburg, 97074 Würzburg, Germany

Correction to: *Scientific Reports* 10.1038/s41598-018-19255-8, published online 16 January 2018

This Article contains an error in the Results section under subheading ‘The clock network in B. *oleae* brain’, where:

“We identified four small (s-LN_v_) and four large (l-LN_v_) ventro-Lateral-Neurons that express PDF, as well as four neurosecretory cells (putative IPCs, Insulin-Producing-Cells; we call them ipc-2 cells according to the naming in *D. melanogaster*) in the pars lateralis (PL).”

should read:

“We identified four small (s-LN_v_) and four large (l-LN_v_) ventro-Lateral-Neurons that express PDF, as well as four neurosecretory cells (putative IPCs, ITP-immunoreactive protocerebral neurons; we call them ipc-2 cells according to the naming in *D. melanogaster*) in the pars lateralis (PL).”

Additionally, this Article contains an error in the legend of Figure 5, where:

“The clock network of *B. oleae*. **(a)** The clock neuropeptide PDF (magenta) is expressed in 4 l-LN_v_ and 4 s-LN_v_, as well as in 4 putative insulin producing cells (ipc-2) in the PL.”

should read:

“The clock network of *B. oleae*. **(a)** The clock neuropeptide PDF (magenta) is expressed in 4 l-LN_v_ and 4 s-LN_v_, as well as in 4 ITP-immunoreactive protocerebral neurons (ipc-2) in the PL.”

