# Supplementary material for: The characterization of the circadian clock in the olive fly Bactrocera oleae (Diptera: Tephritidae) reveals a Drosophila-like organization
Source: Sci Rep. 2018 Jan 16;8:816. doi: 10.1038/s41598-018-19255-8 (PMC5770390; doi:10.1038/s41598-018-19255-8)
Supplement: Supplementary file 1 — Supplementary Information [file 41598_2018_19255_MOESM1_ESM.pdf]

SUPPLEMENTARY INFORMATION

The characterization of the circadian clock in the olive fly *Bactrocera oleae* (Diptera: Tephritidae) reveals a *Drosophila*-like organization

Bertolini Enrico[1,+],
Kistenpfennig Christa[2,+],
Menegazzi Pamela[1],
Keller Alexander[3],
Koukidou Martha[2],
Helfrich-Förster Charlotte[1,*]

[1] Neurobiology and Genetics, Theodor Boveri Institute, Biocentre, University of Würzburg, 97074 Würzburg, Germany
[2] Oxitec Ltd, 71 Milton Park, Oxford, OX14 4RQ, UK
[3] Center for Computation and Theoretical Biology and Department of Bioinformatics, Biocentre, University of Würzburg, 97074 Würzburg, Germany

[*] charlotte.foerster@biozentrum.uni-wuerzburg.de
[+] these authors contributed equally to this work

Supplementary Figure 1

```
CLUSTAL 2.1 multiple sequence alignment - PER

B_ol_per            MEG-ESTESTHNTKVSDSAYSNSCSNSQSQRSGSSKSRLSGSHSSGSSGYGGKPSTQASS 59
B_neohum_per        MEG-ESTESTHNTKVSDSAYSNSCSNSQSQRSGSSKSRLSGSHSSGSSGYGGKPSTQASS 59
D_mel_perA          MEGGESTESTHNTKVSDSAYSNSCSNSQSQRSGSSKSRLSGSHSSGSSGYGGKPSTQASS 60
                    *** ********************************************************

B_ol_per            SDMHIKRVKDKTRKKKKLK------------CSAQTNILENQDDVPNTSEP-KSEEQITL 106
B_neohum_per        SDMHIKRVKDKTRKKKKLK------------CSAQTNILENQDDVPNTSEP-QNEEQIAL 106
D_mel_perA          SDMIIKRNKDKSRKKKKNKGAGQGAGQAQTLISASTSLEGRDEEKPRPSGTGCVEQQICR 120
                    *** *** ***:***** *             **.*.:  .::: *..* .   *:**  

B_ol_per            GCD-----------KAIAQCSRQKENKETSLLLEKETTE-QTKSEISLQFPTPSPLSTAT 154
B_neohum_per        GCD-----------KAIAQCSRQKENKETSLLLEKETTE-QTKSEISLQFPTPSPLSTTT 154
D_mel_perA          ELQDQQHGEDHSEPQAIEQLQQEEEEDQSGSESEADRVEGVAKSEAAQSFPIPSPLSVTI 180
                      :           :** * .:::*:.::.   * : .*  :*** : .** *****.: 

B_ol_per            QQG------------------GKSERTCESAPGKLESSG-----------KAEKLKEESF 185
B_neohum_per        QQG------------------GKSERTCESAPGKLESSG-----------KAEKLKEESF 185
D_mel_perA          VPPSMGGCGGVGHAAGLDSGLAKFDKTWEAGPGKLESMTGVGAAAAGTGQRGERVKEDSF 240
                                         .* ::* *:.******             :.*::**:**

B_ol_per            CCVISMHDGIVLFTTPSITDVLGFPRDMWLGRSFIDFVHPKDRATFASQITTGIPIAESR 245
B_neohum_per        CCVISMHDGIVLFTTPSITDVLGFPRDMWLGRSFIDFVHPKDRATFASQITTGIPIAESR 245
D_mel_perA          CCVISMHDGIVLYTTPSITDVLGYPRDMWLGRSFIDFVHLKDRATFASQITTGIPIAESR 300
                    ************:**********:*************** ********************
```

‡Val243 *Dmel per*^{L}

```
B_ol_per            NSAPKDARSTFCVMLRRYRGLKSGGYGVIGRPVXYEPFRLGLTFREAPEEARPDNNLVSN 305
B_neohum_per        NSAPKDARSTFCVMLRRYRGLKSGGYGVIGRPVSYEPFRLGLTFREAPEEARPDNNLVSN 305
D_mel_perA          GSVPKDAKSTFCVMLRRYRGLKSGGFGVIGRPVSYEPFRLGLTFREAPEEARPDNYMVSN 360
                    .*.****:*****************:******* ********************* :***

B_ol_per            GTNMLLVICATPIKSSYIVPDEILSHKSPKFSIRHTATGLISHVDSAAVSTLGYLPQDLI 365
B_neohum_per        GTNMLLVICATPIKSSYIVPDEILSHKSPKFSIRHTATGLISHVDSAAVSTLGYLPQDLI 365
D_mel_perA          GTNMLLVICATPIKSSYKVPDEILSQKSPKFAIRHTATGIISHVDSAAVSALGYLPQDLI 420
                    ***************** *******:*****:*******:**********:*********

B_ol_per            GRSIFDFYHPEDLMVLKEIYETVMKKGQTAGASFCSKPYRFLIQNGCYILLETEWTSFVN 425
B_neohum_per        GRSIFDFYHPEDLMVLKEIYETVMKKGQTAGASFCSKPYRFLIQNGCYILLETEWTSFVN 425
D_mel_perA          GRSIMDFYHHEDLSVMKETYETVMKKGQTAGASFCSKPYRFLIQNGCYVLLETEWTSFVN 480
                    ****:**** *** *:** *****************************:***********
```

‡Glu464 *per*^{01}

```
B_ol_per            PWSRKLEFVIGHHRVFQGPKHCNVFDSAPANKPKLSDEVRSRNMRIKDEILKLLEESISR 485
B_neohum_per        PWSRKLEFVIGHHRVFQGPKHCNVFDSAPANKPKLSDEVRSRNMRIKDEILKLLEESISR 485
D_mel_perA          PWSRKLEFVVGHHRVFQGPKQCNVFEAAPTCKLKISEEAQSRNTRIKEDIVKRLAETVSR 540
                    *********:**********:****::**: * *:*:*.:*** ***::*:* * *::**

B_ol_per            PSDTVKQEVSRRCQALASFMETLMDEVTRTDLKLELPHENELTVSERDSVMLGEISPHHD 545
B_neohum_per        PSDTVKQEVSRRCQALASFMETLMDEVTRTDLKLELPHENELTVSERDSVMLGEISPHHD 545
D_mel_perA          PSDTVKQEVSRRCQALASFMETLMDEVSRADLKLELPHENELTVSERDSVMLGEISPHHD 600
                    ***************************:*:******************************
```

‡Ser589 *per*^{S}

```
B_ol_per            YYDSKSSTETPPSYNQLNYNENLQRFFNSKPVTAPVEVDPMKNEQSYSISADARNTLSPV 605
B_neohum_per        YYDSKSSTETPPSYNQLNYNENLQRFFNSKPVTAPVEVDPMKNEQSYSISADARSTLSPV 605
D_mel_perA          YYDSKSSTETPPSYNQLNYNENLLRFFNSKPVTAPAELDPPKTEPPEPRGTCVSGASGPM 660
                    *********************** ***********.*:** *.*  . . .: . .: .*:

B_ol_per            Q-CFEGSGGSGSSGNFTSGSHIHMSSITNTSNAGTG------------------------ 640
B_neohum_per        Q-CFEGSGGSGSSGNFTSGSHIHMSSITNTSNAGTG------------------------ 640
D_mel_perA          SPVHEGSGGSGSSGNFTTASNIHMSSVTNTSIAGTGGTGTGTGTGTGTGTGTGTGTGTGT 720
                    .  .*************:.*:*****:**** ****                        
```

```
B_ol_per        -------------------TSSGS--------------AQLVTLTESLLNKHNDEMEKFM 667
B_neohum_per    -------------------TSSGS--------------AQLVTLTESLLNKHNDEMEKFM 667
D_mel_perA      GTGTGTGTGTGTGTGTGNGTNSGTGTGTASSSKGGSAAIPPVTLTESLLNKHNDEMEKFM 780
                                   *.**:                 *******************

B_ol_per        LKKHREARGRCGEKSKK---ATEKVMEYSGPGHGLKRGGSHSWEGDANKPKHSHTNVMDA 724
B_neohum_per    LKKHREARGRCGEKSKK---ATEKVMEYSGPGHGLKRGGSHSWEGDANKPKHSHTNVMDA 724
D_mel_perA      LKKHRESRGRTGEKSKKSANDTLKMLEYSGPGHGIKRGGSHSWEGEANKPKQQLTLGTDA 840
                ******:*** ******    * *::********:**********:*****:. *   **

B_ol_per        QRDYVDHHN--------------------MATNSTSKAYGTLQTTLGETS--------FA 756
B_neohum_per    QRDYVDHHN--------------------MATNSTSKAYGTLQTTLGETS--------FA 756
D_mel_perA      IKGAAGSAGGAVGTGGVGSGGAGVAGGGGSGTGVAGTPEGRATTTSGTGTPGGAGGGGGA 900
                 :. ..  .                     .*. :... *   ** *  :         *

B_ol_per        GSYAAAGLS---------------CTRNVNLWPPFSVGIN--TSHTSQMVAQSGFTPQHG 799
B_neohum_per    GSYAAAGLS---------------CTRNVNLWPPFSVGIN--TSHTSQTVAQSGFTPQHS 799
D_mel_perA      GAAAAAGASSSVGSSTPGPSSYPTCTQNINLWPPFSVGITPPVHSTHTAMAQSSFS-SAG 959
                *: **** *               **:*:**********.  .  *   :***.*: . .

B_ol_per        IFPTFYYIPATAAAAAVAATQTQVKP---NLADMPSTS-------AQALPLQYMTGVMYP 849
B_neohum_per    IFPTFYYIPATAAAAAAAATQAQVKP---NLADMPSTS-------AQALPLQYMTGVMYP 849
D_mel_perA      LFPTFYYIPASLTPTSPTRSPRMHKHPHKGGTDMPTTSQQAAAAAAQAMPLQYMAGVMYP 1019
                :*********: :.:: : :    *    . :***:**       ***:*****:*****

B_ol_per        HPSLFYTHP----ATAMMYQPMSFSNMANSLALSEQ--GNSASAFKTNQPVMLAPTPTKT 903
B_neohum_per    HPSLFYTHP----ATVMMYQPMSFSNMANSLALSEQ--GNSASAFKTNQPCMLAPTPTKT 903
D_mel_perA      HPSLFYTHPAAAAATAMMYQPMPFPGMANALQIPERPLGSQSAYNKSVYTTTPASMTKKV 1079
                *********    **.******.*..***:* :.*:  *..::  *:  .   *. ..*.

B_ol_per        QGAFHSIT-PAQFQRPSSQATSVKAEPGSNMAPSDSSKKGIADS-PIPSVIGDYVSD--- 958
B_neohum_per    QGAFHSIT-PAQLQRPSSQATSVKAEPGSNMAPSDSSKKGIADS-PIPSVIGDYVSD--- 958
D_mel_perA      PGAFHSVTTPAQVQRPSSQSASVKTEPGSSAAVSDPCKKEVPDSSPIPSVMGDYNSDPPC 1139
                 *****:* ***.******::***:****. * **..** :.** *****:*** **

B_ol_per        -QLNPNDLK-PHTDSNANSDDMDGSSFSSFYSSFIKTTDGSDSPQENDKDGKHRKYKVQT 1016
B_neohum_per    -QLNPNDLKQPNTDSNANSDDMDGSSFSSFYSSFIKTTDGSDSPQENDKDGKHRKYKVQT 1017
D_mel_perA      SSSNPANNK-KYTDSNGNSDDMDGSSFSSFYSSFIKTTDGSESPPDTEKDPKHRKLKSMS 1198
                 . ** : *   ****.************************:** :.:** **** *  :

B_ol_per        DSKS--MDNAEEXQTLHGDG 1034
B_neohum_per    DSKS--MDNAEEDQTLHGDG 1035
D_mel_perA      TSESKIMEHPEEDQTQHGDG 1218
                 *:*  *::.** ** ****
```

NLS: nuclear localization signal
PAS-A and PAS-B domain: PER/ARNT/SIM
CLD: cytoplasmic localization domain
CCID: CKL:CYC inhibition domain – not always specified
TG repeats

CLUSTAL 2.1 Multiple Sequence Alignments

Sequence type explicitly set to Protein
Sequence format is Pearson
Sequence 1: B_ol_per   1034 aa
Sequence 2: B_neohum_per   1035 aa
Sequence 3: D_mel_perA   1218 aa

Sequences (1:2) Aligned. Score: 98.55
Sequences (1:3) Aligned. Score: 68.96
Sequences (2:3) Aligned. Score: 68.89

Percent Identity Matrix - created by Clustal2.1

1: B_ol_per	100.00	98.55	71.54
2: B_neohum_per	98.55	100.00	71.64
3: D_mel_perA	71.54	71.64	100.00

Supplementary Figure 2

CLUSTAL 2.1 multiple sequence alignment - CYC

```
B_ol_CYC        ---------MDEVDDETFDDAKSARTSDENRKQNHSEIEKRRRDKMNTYINELSSMIPMC 51
B_curc_CYC      ---------MDEVDDETFDDAKSARTSDENRKQNHSEIEKRRRDKMNTYINELSSMIPMC 51
D_mel_CYC       MEVQEFCENMEEIEDENYDEEKSARTSDENRKQNHSEIEKRRRDKMNTYINELSSMIPMC 60
                         *:*::**.:*: ***************************************

B_ol_CYC        YVVPRKLDKLTVLKYTVQHLRSIRG--SVHPYSGGDYKPSFLSDQELKMLILQASEGFLF 109
B_curc_CYC      YVVPRKLDKLTVLKYTVQHLRSIRG--SVHPYSGGDYKPSFLSDQELKMLILQASEGFLF 109
D_mel_CYC       FAMQRKLDKLTVLRMAVQHLRGIRGSGSLHPFNGSDYRPSFLSDQELKMIILQASEGFLF 120
                :.: *********: :*****.***  *:**:.*.**:***********:**********

B_ol_CYC        VVDCDRGRILYVSESVSQVLNCSQMDLLGQSWFDILHPKDVAKVKEQLSSLDPCPRDRLI 169
B_curc_CYC      VVDCDRGRILYVSESVSQVLNCSQMDLLGQSWFDILHPKDVAKVKEQLSSLDPSPRDRLI 169
D_mel_CYC       VVGCDRGRILYVSDSVSSVLNSTQADLLGQSWFDVLHPKDIGKVKEQLSSLEQCPRERLI 180
                **.**********:***.***.:* *********:*****:.*********: .**:***

B_ol_CYC        DAKXMLPVKTDIPQSLCRLCPGARRSFFCRMKLKSN-NNQIKEESDTSSSSRSSTKRKSK 228
B_curc_CYC      DAKTMLPVKTDIPQSLCRLCPGARRSFFCRMKLKSN-NNQIKEESDTSSSSRSSTKRKSK 228
D_mel_CYC       DAKTMLPVKTDVPQSLCRLCPGARRSFFCRMKLRTASNNQIKEESDTSSSSRSSTKRKSR 240
                *** *******:*********************::  **********************:

B_ol_CYC        LSVDHKYRVIQCTGYLKSWTPIKNEEQDSESEDNLTNHSSLVAIGRIPPNVLESNVPPSL 288
B_curc_CYC      LSGDHKYRVIQCTGYLKSWTPIKNEEQDSESEDNLTNHSSLVAIGRIPPNVLESNVPPSL 288
D_mel_CYC       LTTGHKYRVIQCTGYLKSWTPIKDEDQDADSDEQTTNLSCLVAIGRIPPNVRNSTVPASL 300
                *: .*******************:*:**::*::: ** *.*********** :*.**.**

B_ol_CYC        DNHPNIRHVLFISRHSVDGKFLFIDQRATLVIGFLPQEMLGTSFYDYFHHEDIPALAESH 348
B_curc_CYC      DNHPNIRHVLFISRHSVDGKFLFIDQRATLVIGFLPQEMLGTSFYDYFHHDDVPALAESH 348
D_mel_CYC       DNHPNIRHVLFISRHSGEGKFLFIDQRATLVIGFLPQEILGTSFYEYFHNEDIAALMESH 360
                **************** :********************:******:***::*:.** ***

B_ol_CYC        KMVIQVPEKVTTQVYRFRCKDNTFIQLQSEWRAFKNPWTTDIEYIIAKNTVFL 401
B_curc_CYC      KMVIQVPEKVTTQVYRFRCKDNTFIQLQSEWKAFKNPWTTDIEYIIAKNTVFL 401
D_mel_CYC       KMVMQVPEKVTTQVYRFRCKDNSYIQLQSEWRAFKNPWTSEIDYIIAKNSVFL 413
                ***:******************::*******:*******::*:******:***
```

bHLH domain
PAS-A and PAS-B domain
Dmel cyc0 mutation is a nonsense mutation at K159

```
CLUSTAL 2.1 Multiple Sequence Alignments

Sequence type explicitly set to Protein
Sequence format is Pearson
Sequence 1: B_ol_CYC        401 aa
Sequence 2: B_curc_CYC      401 aa
Sequence 3: D_mel_CYC       413 aa

Sequences (1:2) Aligned. Score: 98.50
Sequences (1:3) Aligned. Score: 82.54
Sequences (2:3) Aligned. Score: 81.80


Percent Identity  Matrix - created by Clustal2.1

1: B_ol_CYC     100.00   98.50   82.54
2: B_curc_CYC    98.50  100.00   81.80
3: D_mel_CYC     82.54   81.80  100.00
```

Supplementary Figure 3

```
CLUSTAL 2.1 multiple sequence alignment - CRY


B_ol_CRY        MAKR-ANVIWFRHGLRFHDNRALLEAISDKTEGIALIPLFIFDGESAGTKTVGYNRMSFL 59
B_tryi_CRY      MAKR-ANVMWFRHGLRLHDNPALLEAISDKTEGIALIPLFIFDGESAGTKTVGYNRMSFL 59
D_mel_CRY       MATRGANVIWFRHGLRLHDNPALLAALADKDQGIALIPVFIFDGESAGTKNVGYNRMRFL 60
                **.* ***:*******:*** *** *::** :******:***********.****** **

B_ol_CRY        LNSLADIDKQLKAIRGANDIAGKLYLFQGNPTTVFRRLNEYFRLNKICFEQDCEPIWNRR 119
B_tryi_CRY      LNSLAEIDKQLKAIRXASDIAGKLYLFQGNPTTVFRRLNEYYRLNKICFEQDCEPIWNRR 119
D_mel_CRY       LDSLQDIDDQLQAAT---DGRGRLLVFEGEPAYIFRRLHEQVRLHRICIEQDCEPIWNER 117
                *:** :**.**:*     *  *:* :*:*:*: :****:*  **::**:*********.*

B_ol_CRY        DDSVRALCNDLGIEAVEKVSHTLWDPRTVISTNGGIPPLTYQMFLHTVEIIGAPPRPVQD 179
B_tryi_CRY      DDSVRALCNDLDIEAVEKVSHTLWDPRTVISTNGRIPPLTXQMFLHTVEIIGAPPRPVED 179
D_mel_CRY       DESIRSLCRELNIDFVEKVSHTLWDPQLVIETNGGIPPLTYQMFLHTVQIIGLPPRPTAD 177
                *:*:*:**.:*.*: ***********: **.*** ***** *******:*** ****. *

B_ol_CRY        PEWDGVKFLKLTDNMLMELNAFWQFPTPEDFNIFPDNINYVAKVKWLGGEQQALLHLAER 239
B_tryi_CRY      PEWDGVEFLKLTDNMLMELNAFWQFPTPEDFNIFPDNISYVAKVKWRGGEQQALXHLAER 239
D_mel_CRY       ARLEDATFVELDPEFCRSLKLFEQLPTPEHFNVYGDNMGFLAKINWRGGETQALLLLDER 237
                .. :.. *::*  ::  .*: * *:****.**:: **:.::**::* *** ***  * **

B_ol_CRY        LKVEERAFKNGYYLPNQANPNILESPKSMSAHLRFGCLSVRRFYWSVHDLFKHVQIEALY 299
B_tryi_CRY      LKVEERAFKNGYYLPNQANPNILESPKSMSAHLRFGCLSVRRFYWSVHDLFKHVQIEAFY 299
D_mel_CRY       LKVEQHAFERGFYLPNQALPNIHDSPKSMSAHLRFGCLSVRRFYWSVHDLFKNVQLRACV 297
                ****::**:.*:****** *** :****************************:**:.*

B_ol_CRY        QRIHMAGGEHITGQLIWREYFYTMSVNNPYYDRMEGNAICLNIPWAPPNQELLQSWRSGQ 359
B_tryi_CRY      HRIHMAGGEHITGQLIWRXYFYTMSVNNPYYDRMEGNEICLNIPWAPPNQEQLQSWRSGQ 359
D_mel_CRY       RGVQMTGGAHITGQLIWREYFYTMSVNNPNYDRMEGNDICLSIPWAKPNENLLQSWRLGQ 357
                : ::*:** ********* ********** ******* ***.**** **:: ***** **

B_ol_CRY        TGFPLIDAAMRQLLAEGWLHHTLRNTVATFLTRGALWQSWEHGLRHFLKYLLDADWSVCA 419
B_tryi_CRY      TGFPLIDAAMRQLLAEGWLHHTLRNTVATFLTRGALWQSWEHGLRHFLKYLLDADWSVCA 419
D_mel_CRY       TGFPLIDGAMRQLLAEGWLHHTLRNTVATFLTRGGLWQSWEHGLQHFLKYLLDADWSVCA 417
                *******.**************************.*********:***************

B_ol_CRY        GNWMWVSSSAFERLLDSSLVSCPIAFSKRLDPKGEYIRQYVPELAKVPQEYIYEPWRMPQ 479
B_tryi_CRY      GNWMWVSSSAFERLLDSSLVSCPIAFSKRLDPKGEYIRQYVPELAKIPQEYIHEPWRMPQ 479
D_mel_CRY       GNWMWVSSSAFERLLDSSLVTCPVALAKRLDPDGTYIKQYVPELMNVPKEFVHEPWRMSA 477
                ********************:**:*::*****.* **:****** ::*:*:::*****.

B_ol_CRY        DMQENYECVIGVQYPERIVDLAKVSKRNTLAMQALRQSLIAGGAPDEGPPHCRPSNEEEV 539
B_tryi_CRY      EMQENYECVIGVQYPERIVDLAKVSKRNTLAXQTLRQSLIAGGAPDEGPPHCRPSNEEEV 539
D_mel_CRY       EQQEQYECLIGVHYPERIIDLSMAVKRNMLAMKSLRNSLITP------PPHCRPSNEEEV 531
                : **:***:***:*****:**: . *** ** ::**:***:       ************

B_ol_CRY        HQFFWLVD--- 547
B_tryi_CRY      HQFFWLVD--- 547
D_mel_CRY       RQFFWLADVVV 542
                :*****.*
```

N-terminal DNA photolyase domain classI (4-195)
C-terminal FAD binding domain (213-516)
classI conserved C-terminal site (360-372)

aa putatively involved in binding of
MTHF (7)
FAD (13)
CPD 13)
FAD+CPD (1)
Dmel cry^{b} missense mutation at D410 (FAD binding domain)

```
CLUSTAL 2.1 Multiple Sequence Alignments

Sequence type explicitly set to Protein
Sequence format is Pearson
Sequence 1: B_ol_CRY        547 aa
Sequence 2: B_try_CRY       547 aa
Sequence 3: D_mel_CRY       542 aa

Sequences (1:2) Aligned. Score: 95.43
Sequences (1:3) Aligned. Score: 72.14
Sequences (2:3) Aligned. Score: 71.40

1: B_ol_CRY    100.00   95.43   73.05
2: B_try_CRY    95.43  100.00   72.30
3: D_mel_CRY    73.05   72.30  100.00
```

Supplementary Figure 4

```
CLUSTAL 2.1 multiple sequence alignment - CLK


B_ol_CLK        MEDESDDKDDTKRKSRNLSEKKRRDQFNTLVNDLSALISTSNRKMDKSTVLKSTIAFLKH 60
B_curc_CLK      --------------------KKRRDQFNTLVNDLSALISTSNRKMDKSTVLKSTIAFLKH 40
D_mel_CLK       MDDESDDKDDTKRKSRNLSEKKRRDQFNSLVNDLSALISTSSRKMDKSTVLKSTIAFLKN 60
                                    ********:************.*****************:

B_ol_CLK        HNEATDRSKVFEIQQDWKPTFLTNDEFTHLMLESLDGFIIVFSGIGSICYASESITPLLG 120
B_curc_CLK      HNEATDRSKVFEIQQDWKPTFLTNDEFTHLMLESLDGFIIVFSGIGSICYASESITPLLG 100
D_mel_CLK       HNEATDRSKVFEIQQDWKPAFLSNDEYTHLMLESLDGFMMVFSSMGSIFYASESITSQLG 120
                *******************:**:***:***********::***.:*** *******. **

B_ol_CLK        YLPSDLVNMTIFDLTYEMDHESLLNIFLNPKPVIEPLQTDINSSNQITFYLHLRRGGIDK 180
B_curc_CLK      YLPSDLVNMTIFDLTYEMDHESLLNIFLNPKPVIEPLQTDINSSNQITFYLHLRRGGIDK 160
D_mel_CLK       YLPQDLYNMTIYDLAYEMDHEALLNIFMNPTPVIEPRQTDISSSNQITFYTHLRRGGMEK 180
                ***.** ****:**:******:*****:**.***** ****.******** ******::*

B_ol_CLK        VDANAYELVKFVGYFRNDVNLDSIQSQ--------NTLALPRIFQMNPSAEVDKKLIFVG 232
B_curc_CLK      VDANAYELVKFVGYFRNDVNLDSIQSQ--------NTLALPRIFQMNPSAEVDKKLIFVG 212
D_mel_CLK       VDANAYELVKFVGYFRNDTNTSTGSSSEVSNGSNGQPAVLPRIFQQNPNAEVDKKLVFVG 240
                ******************.* .: .*.        :. .****** **.*******:***

B_ol_CLK        TGRIQTPQLIREMSAIDPTCNEFTSKHSMEWKFLFLDHRAPPIIGYMPFEVLGTSGYDYY 292
B_curc_CLK      TGRIQTPQLIREMSAIDPTCNEFTSKHSMEWKFLFLDHRVPPIIGYMPFEVLGTSGYDYY 272
D_mel_CLK       TGRVQNPQLIREMSIIDPTSNEFTSKHSMEWKFLFLDHRAPPIIGYMPFEVLGTSGYDYY 300
                ***:*.******** ****.*******************.********************

B_ol_CLK        HFDDLESIVACHEELMQKGECKSCYYRFLTKGQQWIWH---------------------- 330
B_curc_CLK      HFDDLDSIVACHEELMQKGECKSCYYRFLTKGQQWIWLQT-------------------- 312
D_mel_CLK       HFDDLDSIVACHEELRQTGEGKSCYYRFLTKGQQWIWLQTDYYVSYHQFNSKPDYVVCTH 360
                *****:********* *.** ****************

B_ol_CLK        ------------------------------------------------------------
B_curc_CLK      ------------------------------------------------------------
D_mel_CLK       KVVSYAEVLKDSRKEGQKSGNSNSITNNGSSKVIASTGTSSKSASATTTLRDFELSSQNL 420


B_ol_CLK        ------------------------------------------------------------
B_curc_CLK      ------------------------------------------------------------
D_mel_CLK       DSTLLGNSLASLGTETAATSPAVDSSPMWSASAVQPSGSCQINPLKTSRPASSYGNISST 480


B_ol_CLK        ------------------------------------------------------------
B_curc_CLK      ------------------------------------------------------------
D_mel_CLK       GISPKAKRKCYFYNNRGNDSDSTSMSTDSVTSRQSMMTHVSSQSQRQRSHHREHHRENHH 540


B_ol_CLK        ------------------------------------------------------------
B_curc_CLK      ------------------------------------------------------------
D_mel_CLK       NQSHHHMQQQQQHQSQQQQHQQHQQLQQQLQHTVGTPKMVPLLPIASTQIMAGNACQFPQ 600


B_ol_CLK        ------------------------------------------------------------
B_curc_CLK      ------------------------------------------------------------
D_mel_CLK       PAYPIASPQLVAPTFLEPPQYLTAIPMQPVIAPFPVAPVLSPLPVQSQTDMLPDTVVMTP 660


B_ol_CLK        ------------------------------------------------------------
B_curc_CLK      ------------------------------------------------------------
D_mel_CLK       TQSQLQDQLQRKHDELQKLILQQQNELRIVSEQLLLSRYTYLQPMMSMGFAPGNMTAAAV 720


B_ol_CLK        ------------------------------------------------------------
B_curc_CLK      ------------------------------------------------------------
```

```
D_mel_CLK       GNLGASGQRGLNFTGSNAVQPQFNQYGFALNSEQMLNQQDQQMMMQQQQNLHTQHQHNLQ 780

B_ol_CLK        ------------------------------------------------------------
B_curc_CLK      ------------------------------------------------------------
D_mel_CLK       QQHQSHSQLQQHTQQQHQQQQQQQQQQQQQQQQQQQQQQQQQQQQQQQQQQLQLQQQNDIL 840

B_ol_CLK        ------------------------------------------------------------
B_curc_CLK      ------------------------------------------------------------
D_mel_CLK       LREDIDDIDAFLNLSPLHSLGSQSTINPFNSSSNNNNQSYNGGSNLNNGNQNNNNRSSNP 900

B_ol_CLK        ------------------------------------------------------------
B_curc_CLK      ------------------------------------------------------------
D_mel_CLK       PQNNNEDSLLSYMQMATESSPSINFHMGISDDGSETQSEDNKMMHTSGSNLVQQQQQQQQ 960

B_ol_CLK        ------------------------------------------------------------
B_curc_CLK      ------------------------------------------------------------
D_mel_CLK       QQQILQQHQQQSNSFFSSNPFLNSQNQNQNQLPNDLEILPYQMSQEQSQNLFNSPHTAPG 1020

B_ol_CLK        ---
B_curc_CLK      ---
D_mel_CLK       SSQ 1023
```

bHLH domain
PAS-A and PAS-B domain
PAS-B C-terminal conserved region (incomplete)
Q-rich stretches and poly-Q (associated with transcriptional activation)

Supplementary Figure 5

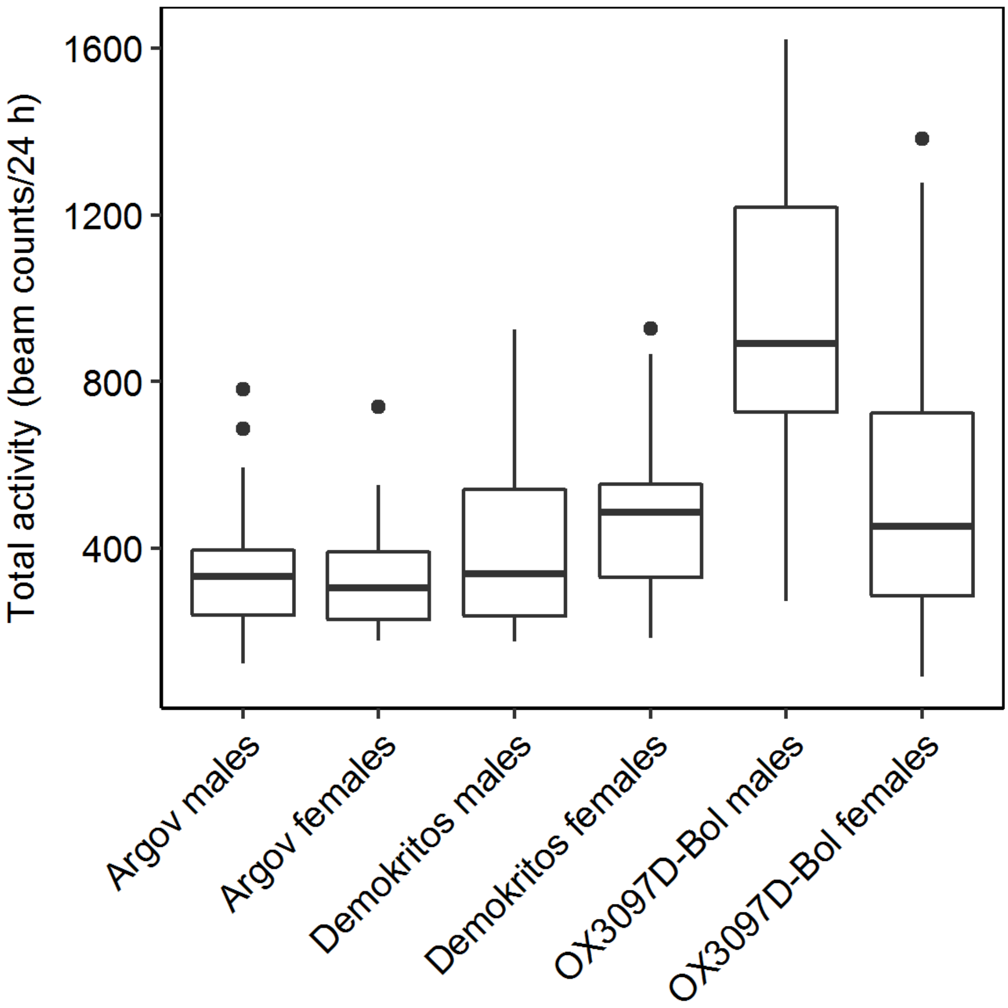

Kruskal-Wallis chi-squared = 56.169, df = 5, p-value = 7.501e-11

	Argov males	Argov females	Demokritos males	Demokritos females	OX3097D-Bol males
Argov females	1.00				
Demokritos males	1.00	1.00			
Demokritos females	0.92	0.38	1.00		
OX3097D-Bol males	0.00	0.00	0.00	0.00	
OX3097D-Bol females	0.07	0.02	0.44	1.00	0.00

t tests with pooled SD (Bonferroni correction)

Supplementary Figure 6

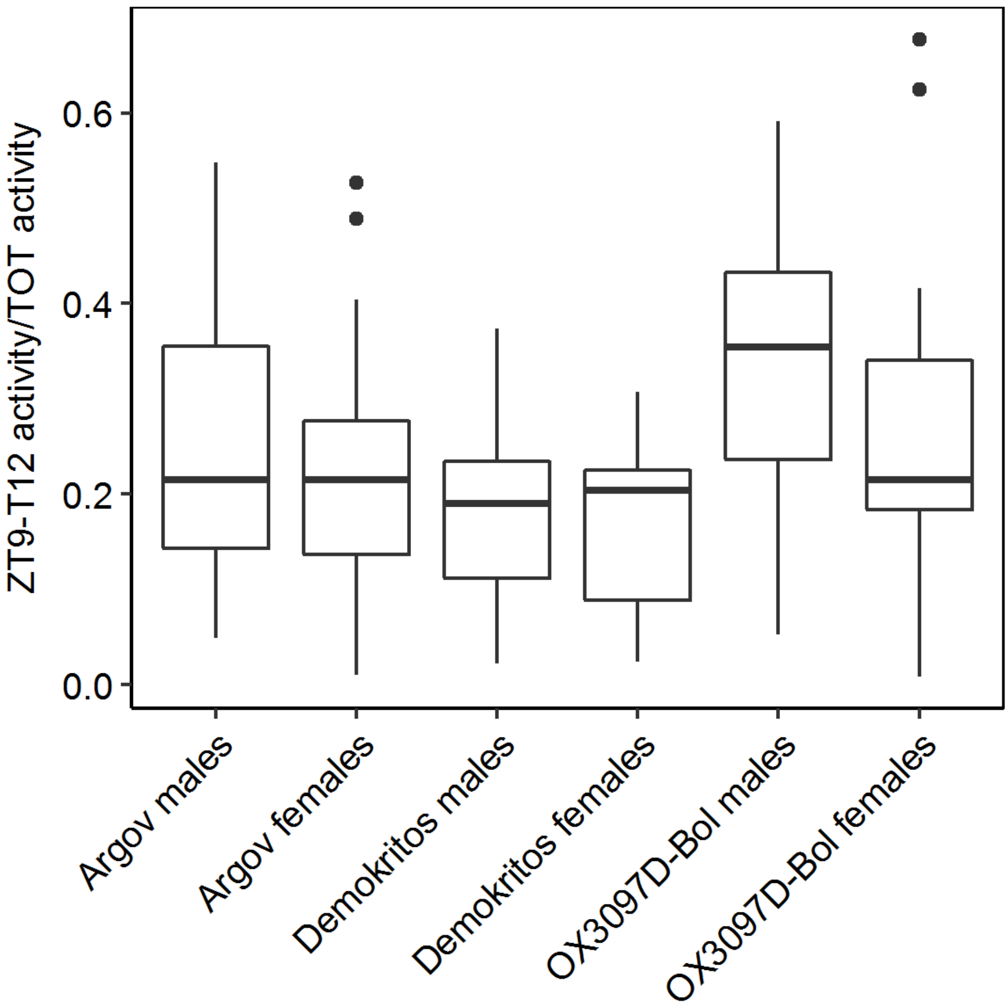

	Df	Sum Sq	Mean Sq	F value	Pr(>F)
group	5	0.55	0.11	6.81	0.0000
Residuals	158	2.55	0.02		

	Argov males	Argov females	Demokritos males	Demokritos females	OX3097D-Bol males
Argov females	1.00				
Demokritos males	0.89	1.00			
Demokritos females	0.50	1.00	1.00		
OX3097D-Bol males	0.10	0.01	0.00	0.00	
OX3097D-Bol females	1.00	1.00	0.57	0.31	0.13

t tests with pooled SD (Bonferroni correction)

Supplementary Figure 7

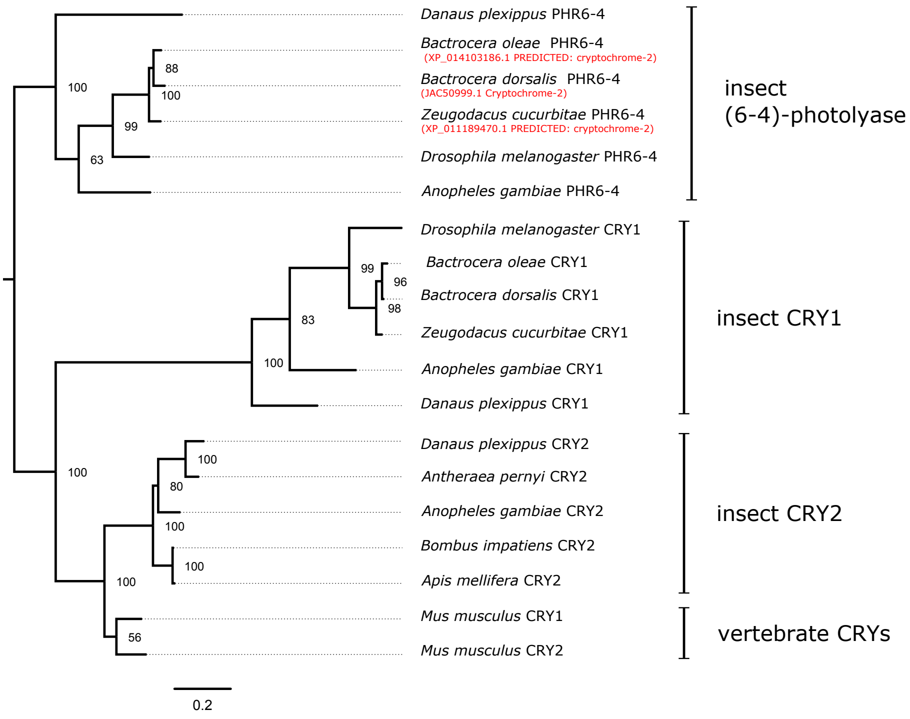

Gene tree of CRY1, CRY2, and insects (6-4)-photolyases reconstructed from amino acids with Maximum Likelihood using RaxML.

The tree shows that sequences annotated in Genbank as “cryptochrome 2“ of *B. oleae* (XP_014103186.1), *B. dorsalis* (JAC50999.1), and *Z. curcubitae* (XP_011189470.1) (highlighted in red) are closely related to insects (6-4)-photolyases. Values at the nodes represent bootstrap values, determined with 100 replicates. GenbankID: *A. gambiae* CRY1 (ABB29886.1), *A. gambiae* CRY2 (ABB29887.1), *A. gambiae* PHR6-4 (EAA10141.3), *A. mellifera* CRY2 (NP_001077099.1), *A. pernyi* CRY2 (ABO38435.1), *B. dorsalis* CRY1 (XP_011206676.1), *B. impatiens* CRY2 (ABO31112.1), *B. oleae* CRY1 (XP_014096643.1), *D. melanogaster* CRY1 (NP_732407.1), *D. melanogaster* PHR6-4 (NP_732407.1), *D. plexippus* CRY1 (AAX58599.1), *D. plexippus* CRY2 (ABA62409.1), *D. plexippus* PHR6-4 (ABO38436.1), *M. musculus* CRY1 (NP_031797.1), *M. musculus* CRY2 (AAD46561.1), *Z. curcubitae* CRY1 (NP_001291679.1).

Supplementary Table 1

gene	gender	interaction	Df	Sum Sq	Mean Sq	F value	Pr(>F)
per	male	timepoint:strain	12	0.19	0.02	1.19	0.3206
Clk	male	timepoint:strain	10	0.13	0.01	1.47	0.1885
cyc	male	timepoint:strain	11	0.26	0.02	1.33	0.2427
cry	male	timepoint:strain	11	0.20	0.02	1.05	0.4200
per	female	timepoint:strain	9	0.09	0.01	0.65	0.7434
Clk	female	timepoint:strain	8	0.11	0.01	0.80	0.6101
cyc	female	timepoint:strain	13	0.35	0.03	1.24	0.2847
cry	female	timepoint:strain	9	0.20	0.02	0.69	0.7165

2-way ANOVA (interaction timepoint:strain)

Supplementary Table 2

Gene/experiment	Oligonucleotide	Sequence (5' → 3')
period		
**identification**	per F1	GGARGGCGAATCAACRGAATCTAC
	per R1	TTGTGCGAGAGTATCTCATCTGGCAC
	per F2	GTGARTTATGAACCCTTCCGTTTGG
	per R2	CTGTAAGACSGTTCRTTCTTCATTGGG
	per F3	GCAAAAGTTCGACGGAAACRCC
	per R3	CMCCAGTCATATATTGMAGCGG
	per F4	GCSGACATGCCGAGCACWTC
	per R4	GGGTTCATAACCAYARCGTCGTCAC
	per 5'RACE	CTCGCCTGCGTTGATGGTTTGCCG
	per 3'RACE	GGCAACGTGACGATCAGTAAGCGGCA
	per 5'RACE nested F	GCCGCTGCTGCCAGATGAGTGACTACC
	per 5'RACE nested R	TTCCCATACAAGTCCACTTTGGCAC
**ORF Argov**	per F5	GTAAAGCGCCAGCAGTTAGTG
	per R5	ATATGCATGTCGCTGCTGCTC
**expression profile**	per qPCR F	GCTGCCGTTGCCGCCACACAGACG
	per qPCR R	CCGAAGGCGCCATATTAGAGCCGGG
cryptochrome		
**identification**	cry F1	TTCAYYGSTGTGCTGTAATATTTGTG
	cry R1	GTKYTTGAAGAGATCATGTACGCTCC
	cry F2	GGCAAATCCMAACATASTYGAATCTCC
	cry R2	CGYTCGGGATACTGCACKCC
	cry 5'RACE	CCAGCAACGCCCGATTGTCGTGG
	cry 3'RACE	CGAGGGCTGGTTGCACCACACGC
**expression profile**	cry qPCR F	CATGGCGAAAGTGCTGGTACC
**ORF Argov**	cry F3	CAGTATGTACCGGAATTGGC
	cry R3	TTTCGGACGATTTGATCACGT
	cry qPCR R	TAGGTCAGTGGCGGGATTCCG
Clock		
**identification**	clk F1	CCNCARYTSATMMGNGARATG
	clk R1	YTGSTRCCADATCCAYTGYTG
	clk 5'RACE	CGTTGCATGTCGGATCTATGGCGG
**expression profile**	clk qPCR F	GGAGGACGAGAGTGACGACAAGG
	clk qPCR R	TCGCTCGGAAGGTAACCCAGCA
cycle		
**identification**	cyc F1	AATCACAGCGARATCGAGAAGCG
	cyc R1	TTGACGGGYAACATGGTCTTYGC
	cyc 5'RACE2	GCACATCGGTATCATGGACGAGAGC
	cyc 3'RACE	GCTTTCTTCGTTAGATCCGTGCCCG
**expression profile**	cyc qPCR F	CTGCAATGGACGAGGTGGACG
	cyc qPCR R	TGCGTCCACGATCACAATCCA
17S rRNA		
**identification**	17S rRNA 5'	MKKYGARGARRTBGCYATYATTCCHAC
	17S rRNA 3'	KGAARTCVAGMWDYTTCAACATVTC
	17S rRNA 3'RACE	CGACGTTCTCGCTCTTCTTCTTGCAGT
**expression profile**	17S rRNA qPCR F	CGTCACTCTCAAGTTAGAGGCATC
	17S rRNA qPCR R	TGTCCTGTTCCAAAGCAGATACAG

Supplementary Table 3

	species	GeneBank ID/reference
Cyc	*Antheraea pernyi*	AAR14937.1
	Bombyx mori	NP_001036982.1
	Danaus plexippus	EHJ64590.1
	Apis mellifera	XP_016770595.1
	Bombus impatiens	XM_012382960.1
	Trachymyrmex zeteki	XM_018454278.1
	Aedes aegypti	JN573265.1
	Anopheles gambiae	Meireles-Filho et al. (2006)
	Anastrepha fraterculus	AQV08515.1
	Zeugodacus cucurbitae	NP_001291680.1
	Drosophila melanogaster	AAC39124.1
	Musca domestica	XM_005180588.3
	Neobellieria bullata	ACJ08742.1
	Mus musculus	AAH25973.1
	Homo sapiens	D89722.1
Per	*Apis cerana*	NP_001315410.1
	Apis mellifera	ARB43935.1
	Camponotus floridanus	XP_011264661.1
	Mythimna separata	AQY60264.1
	Bombyx mori	NP_001036975.1
	Danaus plexippus	AAO48719.1
	Anopheles sinensis	KFB40661.1
	Aedes albopictus	AEX14535.1
	Culex quinquefasciatus	XP_001849299.1
	Ceratitis capitata	ABB20914.1
	Zeugodacus cucurbitae	NP_001291681.1
	Neobellieria bullata	ACJ08740.1
	Musca domestica	AAD39163.1
	Drosophila melanogaster	NP_001259194.1
	Mus musculus	AAC53592.1
	Homo sapiens	NP_073728.1
